# TabPFN-based explainable machine learning for predicting neonatal hyperbilirubinemia: A decade-long retrospective cohort study

**DOI:** 10.1097/MD.0000000000050472

**Published:** 2026-09-04

**Authors:** Yu Zhang, Hui Wang, Liping Zhang

**Affiliations:** a Department of Pediatrics, The Third People’s Hospital of Chengdu, Chengdu, Sichuan, China.

**Keywords:** explainable artificial intelligence, machine learning, neonatal hyperbilirubinemia, prediction model, retrospective cohort, TabPFN, temporal validation

## Abstract

Neonatal hyperbilirubinemia is common in early neonatal life, and delayed recognition of high-risk infants may result in bilirubin-induced neurological injury. Prediction models based on routinely available maternal, perinatal, neonatal, and standardized laboratory variables may support early risk stratification as an adjunct to bilirubin measurement and clinical assessment. This single-center retrospective cohort included 986 neonates admitted between January 1, 2016, and March 1, 2026. The hemolytic-disease variable was excluded before preprocessing and model fitting. Admissions from 2016 through 2023 formed the development cohort (n = 776), and those from 2024 through 2026 formed a held-out temporal validation cohort (n = 210). Stability was assessed using repeated stratified 5-fold cross-validation repeated 5 times. The primary outcome was peak total bilirubin of at least 230 µmol/L during hospitalization. Only predictors documented by the predefined early-admission prediction time were used. Tabular prior-data fitted network (TabPFN) was compared with 5 conventional machine learning models. Performance included discrimination, calibration, Brier score, decision-curve analysis, bootstrap confidence intervals (CIs), and grouped permutation importance. The development and temporal validation cohorts included 468 and 54 events, respectively. Across 25 held-out development folds, TabPFN achieved a mean area under the receiver operating characteristic curve of 0.997 (standard deviation [SD], 0.002), mean area under the precision-recall curve of 0.998 (SD, 0.001), and mean Brier score of 0.023 (SD, 0.009). In temporal validation, TabPFN achieved an area under the receiver operating characteristic curve of 0.999 (95% CI, 0.997–1.000), area under the precision-recall curve of 0.997 (95% CI, 0.991–1.000), and Brier score of 0.013. At the development-derived threshold of 0.538, sensitivity was 1.000, specificity 0.981, positive predictive value 0.947, negative predictive value 1.000, and F1 score 0.973. Random forest had marginally higher temporal discrimination, whereas TabPFN had the lowest Brier score. Leading predictors were maternal disease count, abortion or preterm history, postnatal abnormality, and birth-weight category. After exclusion of the hemolytic-disease variable, TabPFN showed stable repeated-cross-validation performance and strong temporal discrimination, calibration, and clinical net benefit. These single-center findings support TabPFN as a candidate decision-support approach requiring independent multicenter validation and prospective benchmarking against standard-of-care bilirubin risk assessment.

## 1. Introduction

The disease known as newborn hyperbilirubinemia is one of the most frequently encountered clinical disorders during the early neonatal period. A significant majority of neonates experience increased bilirubin levels that are only temporary and disappear without any long-term repercussions. On the other hand, a certain subset of babies may develop extreme hyperbilirubinemia, which necessitates prompt monitoring and treatment. In the event that severe hyperbilirubinemia is not identified and treated in a timely manner, unconjugated bilirubin has the potential to traverse the immature blood–brain barrier, resulting in acute bilirubin encephalopathy or kernicterus, which ultimately leads to irreversible damage to the neuroscience. In light of this, the early identification of newborns who are at an elevated risk of hyperbilirubinemia continues to be an important topic in the field of neonatal care.^[1–3]^

There are a number of maternal, perinatal, neonatal, and institutionally available laboratory-related variables that can affect the development of neonatal hyperbilirubinemia as well as its progression. These factors include gestational age, birth weight, preterm birth, delivery mode, postnatal problems, maternal pregnancy-related disorders, and biochemical abnormalities. Typically, these factors are accessible shortly after birth or during the early stages of hospitalization, although laboratory availability may vary between institutions. Physicians frequently have to interpret these elements concurrently, and it may be challenging to assess their cumulative influence using clinical judgment alone. A trustworthy prediction model built on clearly defined characteristics may assist in early risk stratification, tailored monitoring, and timely intervention. Such a model should be viewed as complementary to, rather than a replacement for, guideline-based bilirubin assessment and clinical judgment.^[4–7]^

For the purpose of determining risk factors and developing clinical prediction tools for neonatal hyperbilirubinemia, traditional statistical models, such as logistic regression (LR), have been utilized extensively. The ability of these models to capture nonlinear correlations and complicated interactions among variables may be restricted, despite the fact that they are simple to interpret due to their simplicity. Machine learning (ML) techniques have opened up new avenues for clinical prediction in recent years. These techniques are able to model high-dimensional interactions and enhance tailored risk estimation, which has led to the creation of new prospects. Medical tabular data have been subjected to the application of a number of ML algorithms, such as random forest, support vector machine (SVM), gradient boosting, and other related ensemble approaches. In spite of this, many standard ML models call for careful hyperparameter tweaking, are sensitive to sample size and data dispersion, and may exhibit unstable performance when applied to clinical datasets that are relatively small or that are only collected from a single site.^[8,9]^

Tabular prior-data fitted network (TabPFN) is a recently developed prior-data fitted network designed for tabular prediction tasks. Unlike conventional ML algorithms that require extensive dataset-specific training and hyperparameter optimization, TabPFN performs in-context prediction by using knowledge learned from large numbers of synthetic tabular tasks. This characteristic may be particularly useful for clinical tabular datasets, where sample sizes are often moderate, and variables are heterogeneous. In addition, combining TabPFN with model-interpretability techniques may provide not only accurate prediction but also clinically meaningful information regarding the relative contribution of key maternal, perinatal, neonatal, and laboratory predictors.^[10]^

However, evidence regarding the application of TabPFN to neonatal hyperbilirubinemia prediction remains limited. Whether a TabPFN-based explainable ML model can provide stable prediction across repeated resampling and later calendar periods using routinely available clinical variables has not been fully investigated. To address this gap, we conducted a decade-long retrospective cohort study to develop the model during an earlier admission period, assess stability using repeated stratified cross-validation, and evaluate locked models in a later temporal validation cohort. TabPFN was compared with conventional ML models, and grouped permutation importance was used to identify influential retained predictors.

## 2. Methods

### 2.1. Study design and data source

This single-center retrospective cohort study was conducted to develop, repeatedly resample, and temporally validate an explainable ML model for predicting neonatal hyperbilirubinemia. Consecutive neonates admitted between January 1, 2016, and March 1, 2026, were screened from the institutional electronic medical record system. Demographic, maternal, perinatal, neonatal, laboratory, and timestamp variables were extracted from routinely collected clinical records. Admissions from January 1, 2016, through December 29, 2023, formed the development period, whereas admissions from January 4, 2024, through February 26, 2026, formed the temporal validation period. During revision, 2 authors re-audited the extraction dictionary, variable coding, timestamps, and table-generation workflow. Because the hemolytic-disease field showed an irreconcilable coding conflict, the field was completely excluded from the predictor matrix before preprocessing, resampling, model fitting, threshold selection, calibration, decision-curve analysis (DCA), and interpretation. The final analysis used de-identified data.

### 2.2. Study population

Neonates with available clinical information, timestamped candidate predictors, and bilirubin outcome data during the index hospitalization were eligible. For neonates with multiple admissions, only the first eligible hospitalization was retained. Records with duplicate identifiers, unavailable outcome information, or variables generated directly from the outcome were excluded. The final cohort included 986 neonates. A chronological split assigned 776 neonates admitted during 2016–2023 to model development and 210 neonates admitted during 2024–2026 to temporal validation. The temporal validation cohort was not used for preprocessing, model selection, threshold selection, or hyperparameter decisions.

### 2.3. Outcome definition

The primary outcome was neonatal hyperbilirubinemia during the index hospitalization, operationally defined as a peak total bilirubin concentration of at least 230 µmol/L; the lowest positive value was 230.0 µmol/L and the highest negative value was 229.0 µmol/L in the audited analytical file. Prediction time zero was defined as completion of the prespecified early-admission assessment window, occurring a median of 4.8 hours after admission (range, 1.6–6.9 hours). Candidate predictors were restricted to information recorded from admission through time zero. Postnatal abnormality status and CK and lactate dehydrogenase (LDH) measurements were documented 0.5 to 6.4 hours before time zero. Outcome ascertainment occurred 12.0 to 71.9 hours after time zero. Peak total bilirubin was used to define and describe the outcome but was never included as a model predictor.

### 2.4. Candidate predictors

Candidate predictors were selected before model development according to clinical relevance, routine availability within the study institution, and availability no later than prediction time zero. The retained predictor set comprised maternal disease count, abortion or preterm history, gestational diabetes mellitus or gestational hypertension, delivery mode, sex, birth-weight category, birth weight, preterm birth, gestational age, postnatal abnormality, creatine kinase (CK) status, CK level, LDH status, and LDH level. The hemolytic-disease variable was completely excluded from the analytical feature matrix before any model operation. CK and LDH were retained because they were complete and institutionally standardized, although they may be selectively ordered or unavailable elsewhere. Feeding status, postnatal weight loss, maternal-neonatal blood-group incompatibility, direct antiglobulin test (DAT) results, prior phototherapy, longitudinal bilirubin trajectories, and genetic factors were unavailable. Patient identifier, group label, and peak total bilirubin were excluded to prevent identifier use and outcome leakage. The missingness, timing relative to prediction time zero, preprocessing, and analytic role for every retained predictor and excluded field were documented.

### 2.5. Data preprocessing

Continuous variables were processed using median imputation and *z*-score normalization, and categorical variables were processed using one-hot encoding. The audited dataset was complete for the retained predictors after recoding. The “No documented abnormality” level denoted an explicitly documented absence of postnatal abnormality, not missing data. For each cross-validation fold, all preprocessing parameters were fitted using only that fold’s training portion and then applied to its held-out portion. For temporal validation, the final preprocessing pipeline was fitted using the entire development cohort and applied unchanged to the temporal cohort. No temporal validation data informed imputation, normalization, encoding, model fitting, or threshold selection.

### 2.6. Model development

TabPFN was the primary model and was fitted with 8 estimators and a fixed random seed. Five prespecified comparators were also fitted: gradient boosting, extra trees, random forest, SVM, and LR. The hemolytic-disease variable was absent from every model input. Within the development period, repeated stratified 5-fold cross-validation repeated 5 times generated 25 held-out predictions per model and 5 out-of-fold predictions per participant. Out-of-fold probabilities were averaged across repeats, and the Youden index applied to these averaged development predictions defined each model’s classification threshold. Final preprocessing pipelines and models were then fitted to the full-development cohort, locked, and evaluated once in the temporal validation cohort.

### 2.7. Model performance, repeated resampling, and temporal validation

Model stability within the development period and performance in temporal validation were assessed using area under the receiver operating characteristic curve (AUROC), area under the precision-recall curve (AUPRC), and Brier score. Accuracy, sensitivity, specificity, positive predictive value (PPV), negative predictive value (NPV), and F1 score were calculated in the temporal cohort at thresholds selected exclusively from averaged out-of-fold development predictions. AUROC and AUPRC 95% confidence intervals (CIs) were estimated with 2000 bootstrap resamples of the temporal cohort. Calibration was evaluated with quantile-based calibration curves and Brier score, and DCA estimated net benefit across clinically relevant threshold probabilities. The temporal cohort remained untouched until the final locked-model evaluation.

### 2.8. Model interpretability

Model-agnostic grouped permutation importance was used to interpret the final TabPFN model in temporal validation. Each of the 14 retained original predictors was permuted independently 10 times; all encoded columns derived from that predictor were varied together, and importance was summarized as the mean decrease in temporal validation AUROC with its standard deviation (SD). The hemolytic-disease field was absent from the fitted model and, therefore, could not contribute to the importance results. Permutation importance was interpreted as a global predictive association rather than a causal effect.

### 2.9. Statistical analysis

Continuous variables were summarized as median and interquartile range (IQR), and categorical variables as number and percentage. Baseline characteristics were compared between outcome groups using the Mann–Whitney *U* test, chi-square test, or Fisher exact test, as appropriate. Development and temporal validation cohorts were compared using absolute standardized mean differences; for categorical variables, the maximum absolute level-specific standardized difference was reported, and values below 0.10 were considered small. Repeated-cross-validation results were summarized as the mean and SD across 25 held-out folds. Temporal validation CIs were obtained by nonparametric bootstrap resampling. A 2-sided *P* value less than.05 was considered statistically significant for descriptive outcome-group comparisons. ML analyses were conducted using Python-based workflows with fixed random seeds.

### 2.10. Ethics statement

This study was approved by the Ethics Committee of Chengdu Third People’s Hospital (approval No. 2026-S-39). Because this was a retrospective study using de-identified routine clinical data, the requirement for written informed consent was waived by the ethics committee. The study was conducted in accordance with the principles of the Declaration of Helsinki and relevant institutional requirements.

## 3. Results

### 3.1. Study population and baseline characteristics

**Figure 1.**
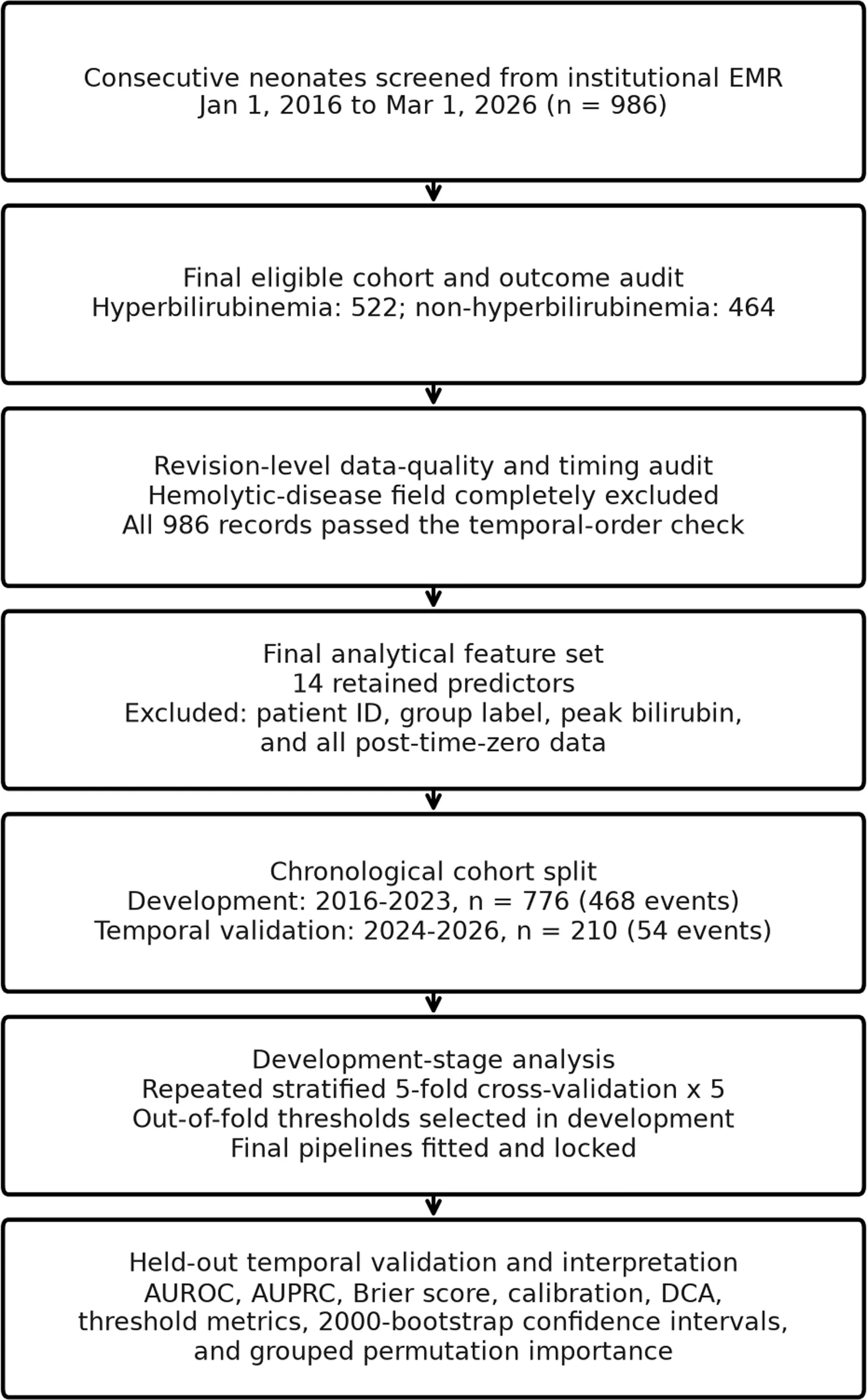
Revised study workflow showing cohort identification, complete exclusion of the hemolytic-disease field, the final 14-predictor feature set, chronological cohort allocation, repeated development-period cross-validation, locked-model fitting, held-out temporal validation, and interpretive analysis. AUPRC = area under the precision-recall curve, AUROC = area under the receiver operating characteristic curve, DCA = decision-curve analysis, EMR = electronic medical record.

**Figure 2.**
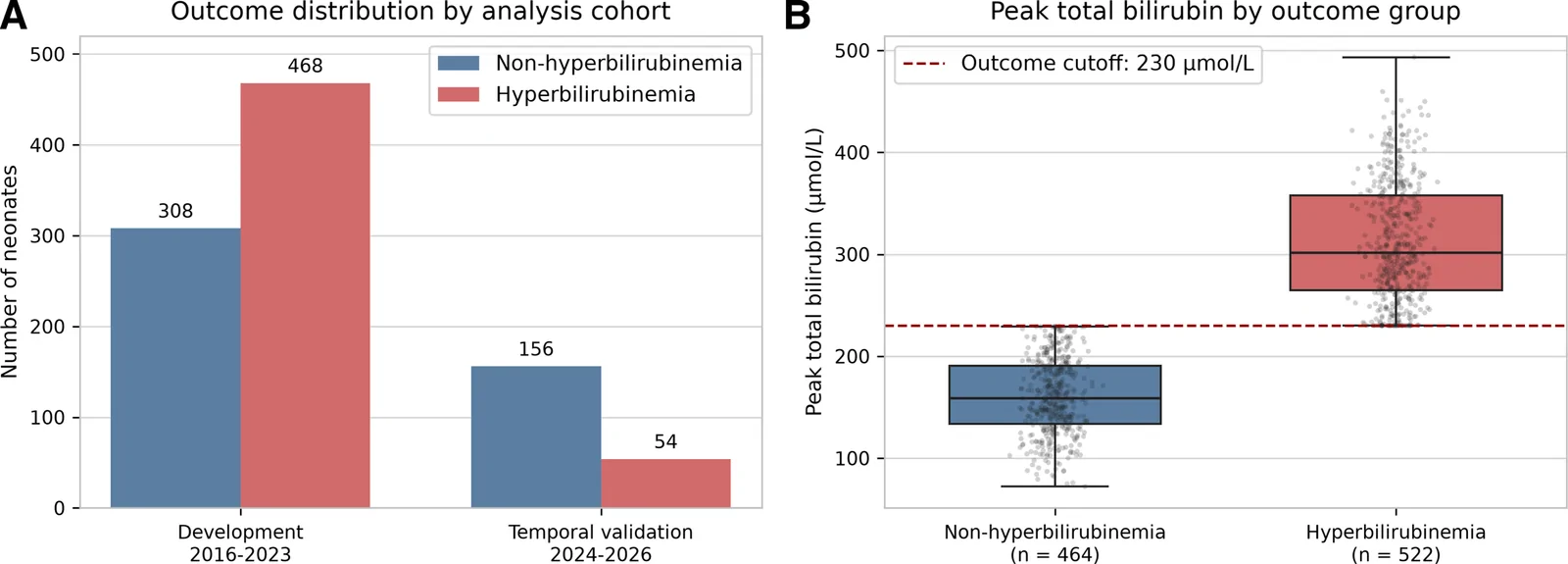
Outcome distribution and outcome definition. Panel A shows counts of neonates with and without hyperbilirubinemia in the development cohort (2016–2023) and held-out temporal validation cohort (2024–2026). Panel B shows peak total bilirubin by outcome group; the dashed horizontal line indicates the operational outcome cutoff of 230 µmol/L.

A total of 986 neonates were included in the final analysis, including 522 neonates with hyperbilirubinemia and 464 neonates without hyperbilirubinemia. The complete revised workflow is presented in Figure 1. Figure 2A shows the outcome distribution in the development and temporal validation cohorts, and Figure 2B shows peak total bilirubin by outcome group together with the prespecified 230 µmol/L cutoff. The median maternal disease count was 2.0 (IQR, 0.0–2.0) in the overall cohort. The median birth weight was 2796.0 g (IQR, 2262.0–3448.8 g), and the median gestational age was 37.1 weeks (IQR, 35.9–39.2 weeks).

Among the 14 retained predictors, neonates with hyperbilirubinemia had a higher maternal disease count (2.0 [IQR, 0.0–2.0] vs 1.0 [IQR, 0.0–2.0], *P* < .001), a lower CK level (183.7 [IQR, 121.0–382.1] U/L vs 216.2 [IQR, 131.2–430.8] U/L, *P* = .013), and a higher LDH level (375.5 [IQR, 184.3–540.5] U/L vs 261.7 [IQR, 135.8–575.6] U/L, *P* = .009). Significant between-group differences were also observed for abortion or preterm history, gestational diabetes mellitus or gestational hypertension, delivery mode, postnatal abnormality, CK status, and LDH status. Birth weight, gestational age, sex, birth-weight category, and preterm birth did not differ significantly. Peak total bilirubin was substantially higher in the hyperbilirubinemia group (301.8 [IQR, 264.6–358.0] µmol/L vs 159.1 [IQR, 133.6–190.4] µmol/L, *P* < .001) but was treated solely as the outcome-defining measurement and is displayed in Figure 2 rather than in the retained-predictor baseline table. The hemolytic-disease field was excluded from all descriptive and model-based analyses. Detailed retained-predictor characteristics are summarized in Table 1.

**Table 1:** Baseline characteristics of retained predictors stratified by neonatal hyperbilirubinemia status.

Variable	Level/statistic	Overall (n = 986)	Non-hyperbilirubinemia (n = 464)	Hyperbilirubinemia (n = 522)	*P* value
maternal_disease_count	Median (IQR)	2.0 (0.0–2.0)	1.0 (0.0–2.0)	2.0 (0.0–2.0)	<.001
birth_weight_g	Median (IQR)	2796.0 (2262.0–3448.8)	2785.5 (2308.8–3500.0)	2814.0 (2209.8–3398.8)	.195
gestational_age_wk	Median (IQR)	37.1 (35.9–39.2)	37.2 (35.9–39.3)	37.0 (36.0–39.0)	.536
creatine_kinase_u_l	Median (IQR)	198.7 (125.3–411.2)	216.2 (131.2–430.8)	183.7 (121.0–382.1)	.013
ldh_u_l	Median (IQR)	307.1 (162.0–555.0)	261.7 (135.8–575.6)	375.5 (184.3–540.5)	.009
abortion_preterm_history	No	487 (49.4)	264 (56.9)	223 (42.7)	<.001
	Yes	499 (50.6)	200 (43.1)	299 (57.3)	
gdm_or_gh	No	641 (65.0)	334 (72.0)	307 (58.8)	<.001
	Yes	345 (35.0)	130 (28.0)	215 (41.2)	
delivery_mode	Cesarean	189 (19.2)	38 (8.2)	151 (28.9)	<.001
	Vaginal	797 (80.8)	426 (91.8)	371 (71.1)	
sex	Female	586 (59.4)	262 (56.5)	324 (62.1)	.085
	Male	400 (40.6)	202 (43.5)	198 (37.9)	
birth_weight_category	2500–3500 g	420 (42.6)	201 (43.3)	219 (42.0)	.097
	<2500 g	347 (35.2)	149 (32.1)	198 (37.9)	
	>3500 g	219 (22.2)	114 (24.6)	105 (20.1)	
preterm	No	535 (54.3)	262 (56.5)	273 (52.3)	.212
	Yes	451 (45.7)	202 (43.5)	249 (47.7)	
postnatal_abnormality	Anemia	162 (16.4)	145 (31.2)	17 (3.3)	<.001
	Anemia + Infection	15 (1.5)	15 (3.2)	0 (0.0)	
	Infection	92 (9.3)	18 (3.9)	74 (14.2)	
	No documented abnormality	717 (72.7)	286 (61.6)	431 (82.6)	
creatine_kinase_status	High	466 (47.3)	247 (53.2)	219 (42.0)	<.001
	Normal	520 (52.7)	217 (46.8)	303 (58.0)	
ldh_status	High	528 (53.5)	209 (45.0)	319 (61.1)	<.001
	Low	179 (18.2)	104 (22.4)	75 (14.4)	
	Normal	279 (28.3)	151 (32.5)	128 (24.5)	

Only the 14 retained predictors are shown. Continuous variables are presented as median (IQR), and categorical variables as n (%). *P* values were calculated using the Mann–Whitney *U* test, chi-square test, or Fisher exact test as appropriate. The hemolytic-disease field was completely excluded. Peak total bilirubin was the outcome-defining measurement and is displayed in Figure 2 rather than treated as a baseline predictor in this table. “No documented abnormality” denotes an explicitly documented absence of postnatal abnormality, not missing data.

IQR = interquartile range.

### 3.2. Development and temporal validation cohorts

The chronological split produced a development cohort of 776 neonates, including 468 with hyperbilirubinemia (60.3%), and a temporal validation cohort of 210 neonates, including 54 with hyperbilirubinemia (25.7%). The development period extended from January 2016 through December 2023, and the temporal validation period extended from January 2024 through February 2026. Table 2 documents the resulting calendar-time distribution shift. The absolute standardized mean difference was 0.699 for outcome prevalence, 0.347 for maternal disease count, 0.282 for postnatal abnormality, 0.253 for gestational diabetes or hypertension, and 0.249 for delivery mode; most remaining retained predictors showed smaller differences. This later-period shift provided a clinically relevant test of calibration and locked-threshold performance. The temporal cohort was held out from every development decision.

**Table 2:** Development and temporal validation cohort characteristics.

Variable	Level/statistic	Development cohort (n = 776)	Temporal validation cohort (n = 210)	Absolute standardized mean difference
hyperbilirubinemia	Yes, n (%)	468 (60.3)	54 (25.7)	0.699
maternal_disease_count	Median (IQR)	2.0 (0.0–2.0)	1.0 (0.0–2.0)	0.347
birth_weight_g	Median (IQR)	2811.5 (2262.0–3414.8)	2770.5 (2263.2–3515.5)	0.024
gestational_age_wk	Median (IQR)	37.2 (36.0–39.1)	37.0 (35.8–39.2)	0.088
creatine_kinase_u_l	Median (IQR)	199.0 (126.8–417.0)	182.0 (119.4–385.6)	0.078
ldh_u_l	Median (IQR)	322.5 (170.6–549.4)	283.2 (141.1–576.4)	0.061
abortion_preterm_history	No	376 (48.5)	111 (52.9)	0.088
	Yes	400 (51.5)	99 (47.1)	
gdm_or_gh	No	485 (62.5)	156 (74.3)	0.253
	Yes	291 (37.5)	54 (25.7)	
delivery_mode	Cesarean	164 (21.1)	25 (11.9)	0.249
	Vaginal	612 (78.9)	185 (88.1)	
sex	Female	454 (58.5)	132 (62.9)	0.089
	Male	322 (41.5)	78 (37.1)	
birth_weight_category	2500–3500 g	341 (43.9)	79 (37.6)	0.133
	<2500 g	272 (35.1)	75 (35.7)	
	>3500 g	163 (21.0)	56 (26.7)	
preterm	No	429 (55.3)	106 (50.5)	0.096
	Yes	347 (44.7)	104 (49.5)	
postnatal_abnormality	Anemia	109 (14.0)	53 (25.2)	0.282
	Anemia + Infection	7 (0.9)	8 (3.8)	
	Infection	82 (10.6)	10 (4.8)	
	No documented abnormality	578 (74.5)	139 (66.2)	
creatine_kinase_status	High	371 (47.8)	95 (45.2)	0.052
	Normal	405 (52.2)	115 (54.8)	
ldh_status	High	425 (54.8)	103 (49.0)	0.114
	Low	134 (17.3)	45 (21.4)	
	Normal	217 (28.0)	62 (29.5)	

Continuous variables are presented as median (IQR), and categorical variables as n (%). Absolute standardized mean differences compare the development period (2016–2023) with the later temporal validation period (2024–2026); values below 0.10 are generally considered small. For categorical variables, the reported value is the maximum absolute level-specific standardized mean difference. Temporal validation was intended to preserve calendar-time distribution shift rather than create balanced cohorts. The hemolytic-disease field was completely excluded.

IQR = interquartile range.

### 3.3. Predictor availability and preprocessing

Complete data were available after recoding for all 14 retained predictors. Continuous predictors were maternal disease count, birth weight, gestational age, CK level, and LDH level. Categorical predictors were abortion or preterm history, gestational diabetes mellitus or gestational hypertension, delivery mode, sex, birth-weight category, preterm birth, postnatal abnormality, CK status, and LDH status. Table 3 confirms that all retained information was available no later than prediction time zero: postnatal abnormality was recorded 0.5 to 6.4 hours before time zero, CK 0.5 to 6.2 hours before time zero, and LDH 0.5 to 5.9 hours before time zero. All 986 records passed the temporal-order audit. The hemolytic-disease field was removed before preprocessing and never entered any model. Peak total bilirubin, group label, patient identifier, and all post-time-zero variables were excluded from model development.

**Table 3:** Retained predictors and excluded fields: missingness, timing, preprocessing, and analytic role.

Variable	Type	Missing, n (%)	Availability vs time zero	Encoding/handling	Role in analysis
maternal_disease_count	Continuous	0 (0.0)	By time zero	Median + *z*-score (fold-specific)	Retained predictor
abortion_preterm_history	Categorical	0 (0.0)	By time zero	One-hot encoding (fold-specific)	Retained predictor
gdm_or_gh	Categorical	0 (0.0)	By time zero	One-hot encoding (fold-specific)	Retained predictor
delivery_mode	Categorical	0 (0.0)	By time zero	One-hot encoding (fold-specific)	Retained predictor
sex	Categorical	0 (0.0)	By time zero	One-hot encoding (fold-specific)	Retained predictor
birth_weight_category	Categorical	0 (0.0)	By time zero	One-hot encoding (fold-specific)	Retained predictor
birth_weight_g	Continuous	0 (0.0)	By time zero	Median + *z*-score (fold-specific)	Retained predictor
preterm	Categorical	0 (0.0)	By time zero	One-hot encoding (fold-specific)	Retained predictor
gestational_age_wk	Continuous	0 (0.0)	By time zero	Median + *z*-score (fold-specific)	Retained predictor
postnatal_abnormality	Categorical	0 (0.0)	0.5–6.4 h before time zero	One-hot; defined “No abnormality” level	Retained predictor
creatine_kinase_status	Categorical	0 (0.0)	0.5–6.2 h before time zero	One-hot encoding (fold-specific)	Retained predictor (institution-specific)
creatine_kinase_u_l	Continuous	0 (0.0)	0.5–6.2 h before time zero	Median + *z*-score (fold-specific)	Retained predictor (institution-specific)
ldh_status	Categorical	0 (0.0)	0.5–5.9 h before time zero	One-hot encoding (fold-specific)	Retained predictor (institution-specific)
ldh_u_l	Continuous	0 (0.0)	0.5–5.9 h before time zero	Median + *z*-score (fold-specific)	Retained predictor (institution-specific)
hemolytic_disease	Excluded field	0 (0.0)	Not used	No preprocessing or encoding	Completely excluded before all model operations
peak_total_bilirubin_umol_l	Outcome	0 (0.0)	12.0–71.9 h after time zero	Outcome definition only	Excluded: outcome leakage
group_label	Outcome-derived	0 (0.0)	Defined after outcome ascertainment	Not encoded	Excluded: outcome-derived
patient_id	Identifier	0 (0.0)	Available at admission	Not encoded	Excluded: identifier

Fourteen predictors were retained. Preprocessing was fitted within each development training fold; the final development-fitted pipeline was applied unchanged to temporal validation. The hemolytic-disease field was completely excluded. Patient identifier, group label, peak total bilirubin, and post-time-zero information were excluded from predictors. All 986 records passed the temporal-order audit.

### 3.4. Repeated cross-validation within the development period

Across the 25 held-out folds generated by repeated stratified 5-fold cross-validation in the development period, TabPFN achieved a mean AUROC of 0.997 (SD, 0.002), a mean AUPRC of 0.998 (SD, 0.001), and a mean Brier score of 0.023 (SD, 0.009). The corresponding mean AUROC, AUPRC, and Brier score were 0.946, 0.952, and 0.087 for gradient boosting; 0.996, 0.997, and 0.024 for extra trees; 0.993, 0.995, and 0.039 for random forest; 0.963, 0.974, and 0.073 for SVM; and 0.836, 0.866, and 0.157 for LR.

Averaged out-of-fold predictions across the 5 repeats were used to select thresholds without reference to the temporal data. The resulting thresholds were 0.538 for TabPFN, 0.570 for gradient boosting, 0.715 for extra trees, 0.640 for random forest, 0.730 for SVM, and 0.574 for LR. These thresholds and the full-development-fitted models were locked before temporal validation.

### 3.5. Temporal validation, calibration, and threshold performance

**Figure 3.**
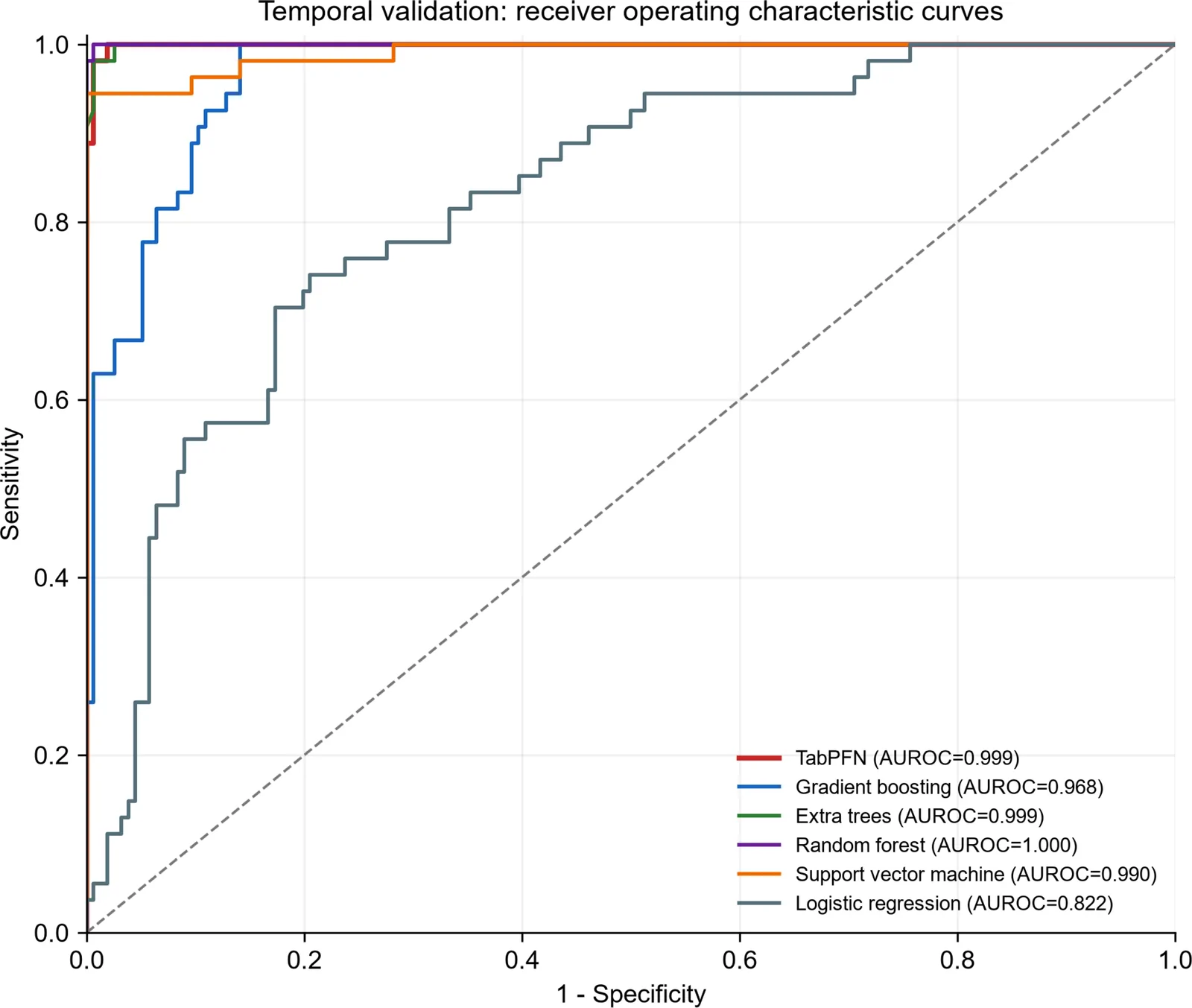
Receiver operating characteristic curves for TabPFN and comparator models in the held-out temporal validation cohort. AUROC = area under the receiver operating characteristic curve, TabPFN = tabular prior-data fitted network.

**Figure 4.**
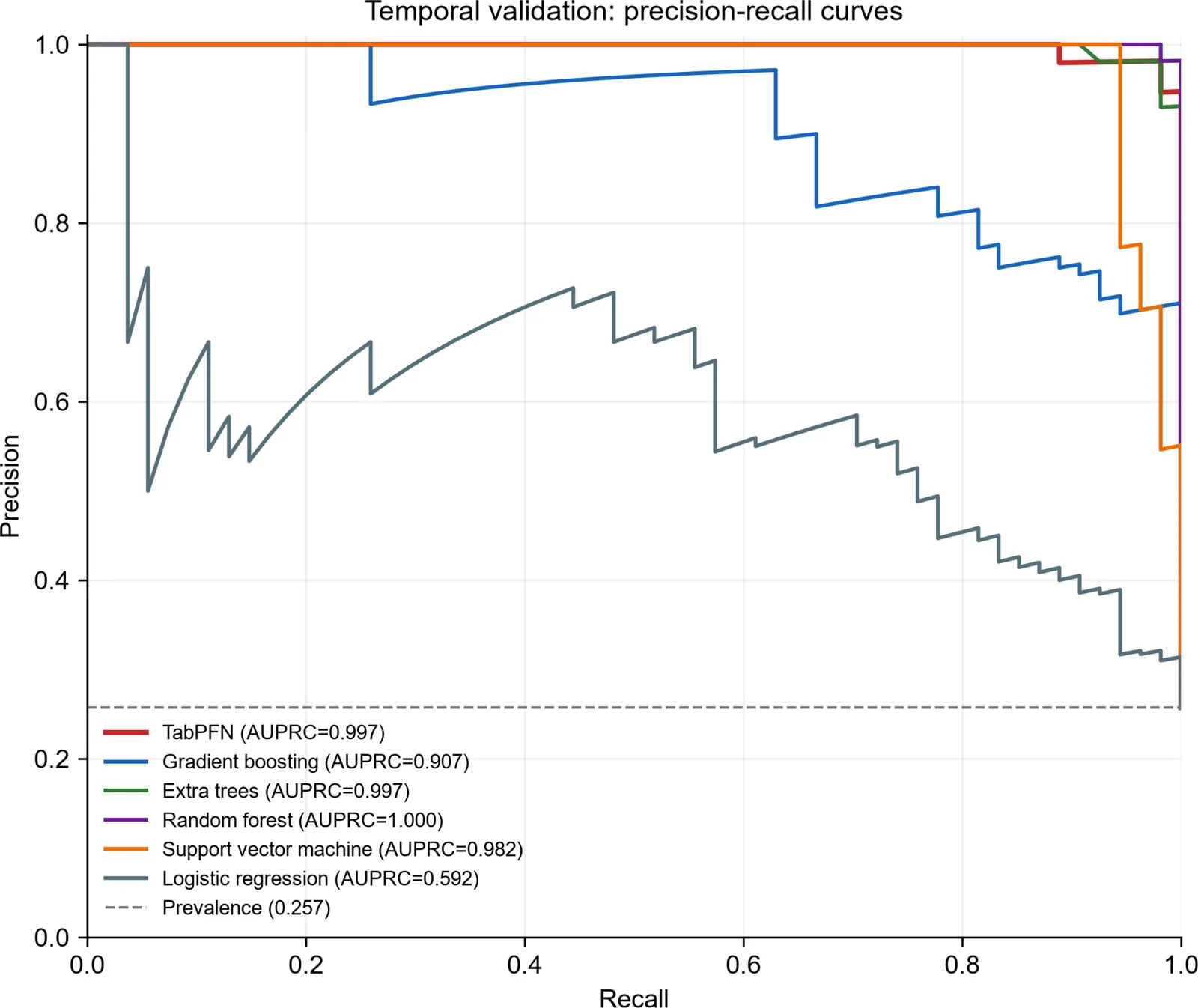
Precision-recall curves for TabPFN and comparator models in the held-out temporal validation cohort. AUPRC = area under the precision-recall curve, TabPFN = tabular prior-data fitted network.

**Figure 5.**
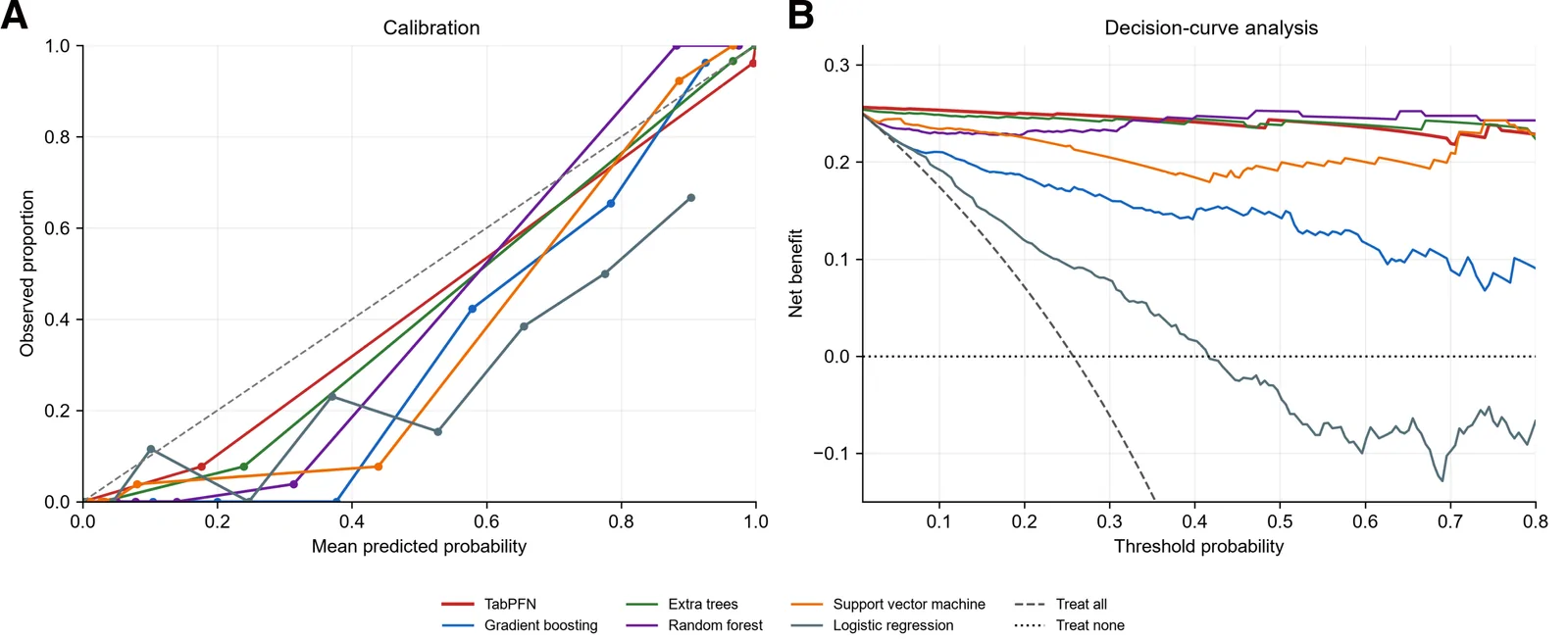
(A) Calibration curves and (B) decision-curve analysis for all evaluated models in the held-out temporal validation cohort. Calibration was summarized across quantile-based probability groups, and decision-curve analysis estimated net benefit across threshold probabilities. TabPFN = tabular prior-data fitted network.

In the temporal validation cohort, random forest had the highest discrimination (AUROC, 1.000; AUPRC, 1.000), whereas TabPFN had the lowest Brier score (0.013) and achieved an AUROC of 0.999 (95% CI, 0.997–1.000) and AUPRC of 0.997 (95% CI, 0.991–1.000). Extra trees achieved an AUROC of 0.999 and AUPRC of 0.997; SVM, 0.990 and 0.982; gradient boosting, 0.968 and 0.907; and LR, 0.822 and 0.592, respectively. Full metrics are presented in Table 4, receiver operating characteristic and precision-recall curves in Figures 3 and 4, and calibration and decision-curve results in Figure 5. TabPFN maintained close agreement between predicted and observed risk and positive net benefit across a wide range of thresholds.

**Table 4:** Temporal validation performance of TabPFN and comparator machine learning models.

Model	AUROC (95% CI)	AUPRC (95% CI)	Brier score	Threshold	Accuracy	Sensitivity	Specificity	PPV	NPV	F1 score
TabPFN	0.999 (0.997–1.000)	0.997 (0.991–1.000)	0.013	0.538	0.986	1.000	0.981	0.947	1.000	0.973
Gradient boosting	0.968 (0.945–0.985)	0.907 (0.832–0.959)	0.092	0.570	0.895	0.907	0.891	0.742	0.965	0.817
Extra trees	0.999 (0.997–1.000)	0.997 (0.991–1.000)	0.014	0.715	0.990	0.981	0.994	0.981	0.994	0.981
Random forest	1.000 (0.999–1.000)	1.000 (0.998–1.000)	0.019	0.640	0.990	0.981	0.994	0.981	0.994	0.981
Support vector machine	0.990 (0.976–1.000)	0.982 (0.955–1.000)	0.042	0.730	0.981	0.944	0.994	0.981	0.981	0.962
Logistic regression	0.822 (0.758–0.882)	0.592 (0.467–0.726)	0.191	0.574	0.733	0.759	0.724	0.488	0.897	0.594

All models were retrained after complete exclusion of the hemolytic-disease variable. Thresholds were selected from averaged out-of-fold predictions generated by repeated stratified 5-fold cross-validation repeated 5 times in the development period (2016–2023). Metrics were evaluated once in the held-out temporal validation cohort (2024–2026; n = 210). AUROC and AUPRC 95% CIs were estimated using 2000 bootstrap resamples.

AUPRC = area under the precision-recall curve, AUROC = area under the receiver operating characteristic curve, CI = confidence interval, NPV = negative predictive value, PPV = positive predictive value, TabPFN = tabular prior-data fitted network.

Threshold analysis in the temporal cohort showed that TabPFN maintained 100% sensitivity and 100% NPV from thresholds 0.10 through 0.50. At thresholds of 0.10, 0.20, and 0.30, specificity was 0.955, 0.962, and 0.968, respectively. At the locked Youden-derived threshold of 0.538, accuracy was 0.986, sensitivity 1.000, specificity 0.981, PPV 0.947, NPV 1.000, F1 score 0.973, and 27.1% of neonates were classified as high risk. Detailed threshold results are shown in Table 5.

**Table 5:** Threshold-dependent performance of the final TabPFN model in temporal validation.

Threshold rule	Threshold	Sensitivity	Specificity	PPV	NPV	Accuracy	F1 score	Predicted high-risk, %
0.10	0.100	1.000	0.955	0.885	1.000	0.967	0.939	29.0
0.20	0.200	1.000	0.962	0.900	1.000	0.971	0.947	28.6
0.30	0.300	1.000	0.968	0.915	1.000	0.976	0.956	28.1
0.40	0.400	1.000	0.968	0.915	1.000	0.976	0.956	28.1
0.50	0.500	1.000	0.981	0.947	1.000	0.986	0.973	27.1
Youden	0.538	1.000	0.981	0.947	1.000	0.986	0.973	27.1

Candidate-threshold metrics were calculated in the held-out temporal validation cohort. The Youden threshold (0.538) was selected from averaged out-of-fold development predictions generated by repeated stratified 5-fold cross-validation repeated 5 times and was locked before temporal evaluation.

NPV = negative predictive value, PPV = positive predictive value, TabPFN = tabular prior-data fitted network.

### 3.6. Model interpretability

**Figure 6.**
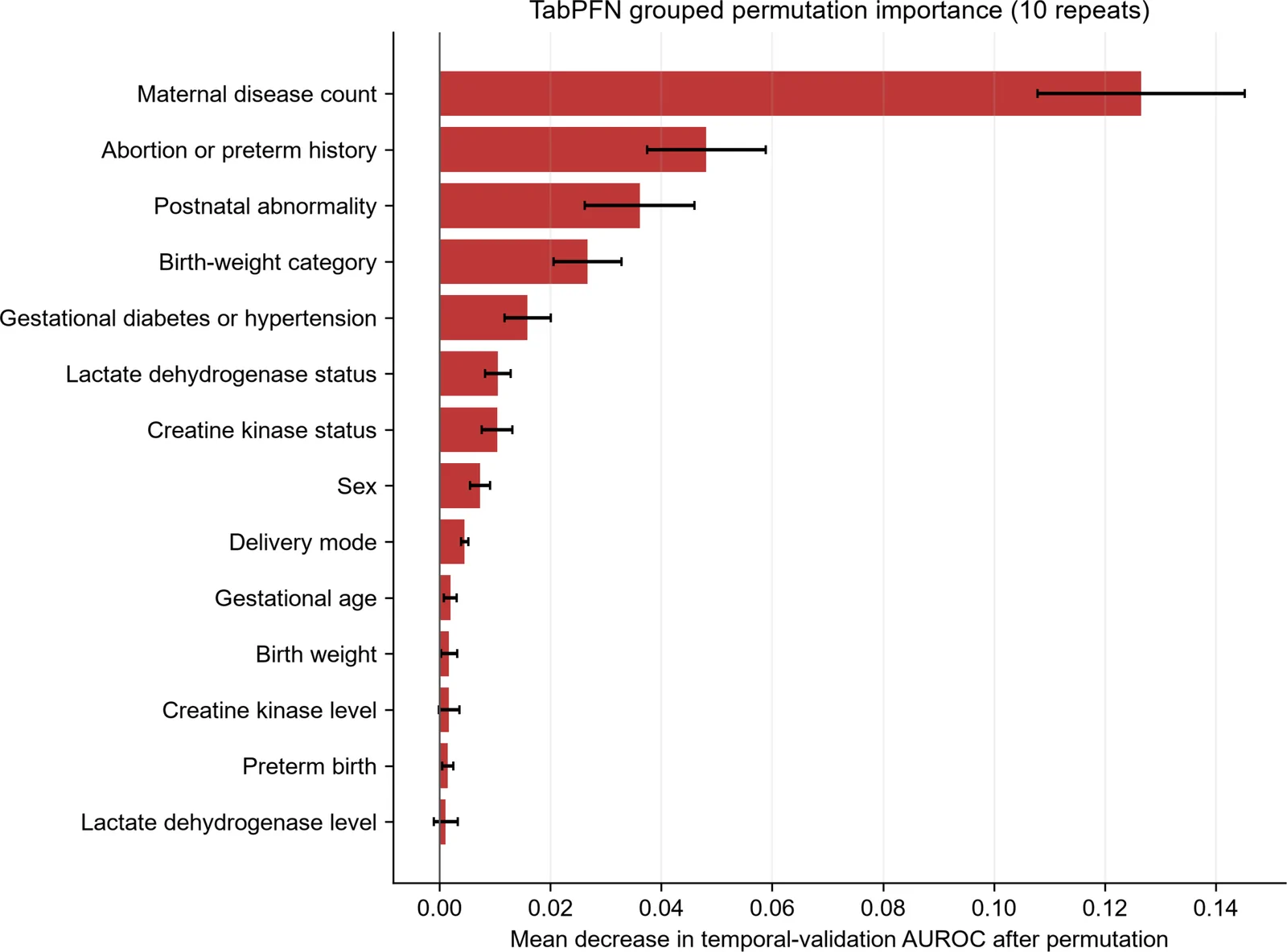
Grouped permutation importance for the final TabPFN model in temporal validation. Each retained predictor was permuted independently 10 times, and bars show the mean decrease in AUROC with standard deviation error bars. The hemolytic-disease variable is absent because it was completely excluded before model fitting. AUROC = area under the receiver operating characteristic curve, TabPFN = tabular prior-data fitted network.

Before permutation, the final TabPFN model had a temporal validation AUROC of 0.9991. Across 10 grouped permutations per predictor, maternal disease count produced the largest mean AUROC decrease (0.1265 [SD, 0.0187]), followed by abortion or preterm history (0.0481 [SD, 0.0107]), postnatal abnormality (0.0361 [SD, 0.0099]), and birth-weight category (0.0267 [SD, 0.0061]). Smaller contributions were observed for gestational diabetes or hypertension, LDH status, CK status, sex, delivery mode, gestational age, birth weight, CK level, preterm birth, and LDH level. The hemolytic-disease variable was absent from the fitted model and importance analysis. Results are shown in Figure 6 and Table 6.

**Table 6:** Grouped permutation importance for retained predictors in the final TabPFN model.

Feature	Importance metric	Baseline AUROC	Mean permuted AUROC	Mean AUC drop (SD)
Maternal disease count	Grouped permutation AUC drop, 10 repeats	0.9991	0.8725	0.1265 (0.0187)
Abortion or preterm history	Grouped permutation AUC drop, 10 repeats	0.9991	0.9509	0.0481 (0.0107)
Postnatal abnormality	Grouped permutation AUC drop, 10 repeats	0.9991	0.9629	0.0361 (0.0099)
Birth-weight category	Grouped permutation AUC drop, 10 repeats	0.9991	0.9724	0.0267 (0.0061)
Gestational diabetes or hypertension	Grouped permutation AUC drop, 10 repeats	0.9991	0.9832	0.0159 (0.0042)
Lactate dehydrogenase status	Grouped permutation AUC drop, 10 repeats	0.9991	0.9885	0.0105 (0.0023)
Creatine kinase status	Grouped permutation AUC drop, 10 repeats	0.9991	0.9887	0.0104 (0.0027)
Sex	Grouped permutation AUC drop, 10 repeats	0.9991	0.9917	0.0073 (0.0018)
Delivery mode	Grouped permutation AUC drop, 10 repeats	0.9991	0.9945	0.0045 (0.0006)
Gestational age	Grouped permutation AUC drop, 10 repeats	0.9991	0.9971	0.0020 (0.0011)
Birth weight	Grouped permutation AUC drop, 10 repeats	0.9991	0.9973	0.0017 (0.0015)
Creatine kinase level	Grouped permutation AUC drop, 10 repeats	0.9991	0.9974	0.0017 (0.0019)
Preterm birth	Grouped permutation AUC drop, 10 repeats	0.9991	0.9976	0.0015 (0.0010)
Lactate dehydrogenase level	Grouped permutation AUC drop, 10 repeats	0.9991	0.9979	0.0011 (0.0022)

Importance was evaluated in the held-out temporal validation cohort after complete exclusion of the hemolytic-disease variable. Each original predictor was permuted independently 10 times, with all encoded columns derived from that predictor varied together. Results are the mean decrease in AUROC and SD across repeats.

AUC = area under the curve, AUROC = area under the receiver operating characteristic curve, SD = standard deviation, TabPFN = tabular prior-data fitted network.

## 4. Discussion

### 4.1. Principal findings

In this decade-long, single-center cohort of 986 neonates, we developed the models in an earlier admission period, assessed internal stability with repeated stratified cross-validation, and evaluated locked models in a later temporal cohort after completely excluding the hemolytic-disease variable. TabPFN remained highly stable across 25 development folds and achieved temporal AUROC 0.999, AUPRC 0.997, and the lowest Brier score of 0.013. Random forest had marginally higher temporal discrimination, so the revised results do not claim that TabPFN was uniformly superior. At the locked TabPFN threshold, sensitivity and NPV were both 1.000. Grouped permutation importance identified maternal disease count, abortion or preterm history, postnatal abnormality, and birth-weight category as the leading retained predictors.

The revised timestamp audit confirmed that all retained predictors preceded outcome ascertainment in all 986 records. Prediction time zero occurred within the early-admission period, predictor information was limited to data available by that time, and outcome ascertainment occurred at least 12 hours later. Peak total bilirubin was used only to define and describe the outcome and was excluded from the predictor set. These design changes reduce, although they cannot eliminate, concerns about temporal and outcome leakage.

### 4.2. Interpretation of model performance

TabPFN, extra trees, random forest, and SVM all showed strong nonlinear predictive performance, whereas LR performed less well. This pattern is plausible because neonatal hyperbilirubinemia may reflect interactions among maternal disease burden, perinatal background, neonatal physiological maturity, early postnatal status, and biochemical abnormalities. The prior-data fitted structure of TabPFN may efficiently combine heterogeneous tabular information, but the near-perfect temporal discrimination of random forest and extra trees demonstrates that the signal was not unique to TabPFN.^[11–17]^

TabPFN had the lowest temporal Brier score, supporting favorable overall probability accuracy despite the marked decline in outcome prevalence from the development to temporal period. This is clinically relevant because overestimation can cause unnecessary monitoring, whereas underestimation can delay evaluation. Nevertheless, strong single-center temporal performance does not establish transportability, and calibration should be reassessed in external populations with different bilirubin screening practices and case mix.

### 4.3. Clinical implications

The threshold analysis provides clinically interpretable operating points. At thresholds of 0.10 to 0.50, TabPFN achieved 100% sensitivity and NPV in temporal validation, suggesting possible screening value when missed cases are the primary concern. At the development-derived threshold of 0.538, specificity was 0.981 and PPV 0.947, with 27.1% of neonates classified as high risk. These findings remain exploratory and should not be used to guide treatment until independently validated against guideline-based risk assessment.

A future electronic implementation could generate a risk estimate after completion of the early-admission predictor window. Such a system could not replace bilirubin measurement, age-specific guideline assessment, or clinical judgment. The current temporal validation supports further evaluation but remains internal to one hospital; implementation would require external calibration, prospective impact assessment, governance review, and monitoring for performance drift.

### 4.4. Model interpretability

Interpretability is essential for clinical ML applications. Repeated grouped permutation showed that maternal disease count was the leading retained predictor, followed by abortion or preterm history, postnatal abnormality, and birth-weight category. Maternal disease burden may reflect pregnancy-related complications affecting neonatal adaptation. A history of abortion or preterm birth may capture maternal or placental risk, while postnatal abnormality may reflect early neonatal instability, infection, feeding difficulty, or other disorders that influence bilirubin production and clearance. The birth-weight category may add nonlinear information beyond continuous birth weight. These rankings describe global predictive contribution in the temporal cohort and should not be interpreted as causal effects. The hemolytic-disease field was not interpreted because it was completely excluded before model fitting.

CK and LDH contributed less than the leading predictors in the revised temporal permutation analysis. They were retained because they were complete and routinely collected in this institution, but they may be unavailable or selectively ordered elsewhere. External validation should therefore test the model both with and without institution-specific laboratory markers and should examine whether predictor importance changes across clinical settings.

### 4.5. *Comparison with conventional* ML *approaches*

Conventional ensemble models also performed strongly. Random forest achieved the highest temporal AUROC and AUPRC, while extra trees closely matched TabPFN. TabPFN’s principal relative advantage was its lower Brier score and perfect sensitivity and NPV at the locked threshold. Accordingly, the revised manuscript no longer claims uniform superiority. The results instead support TabPFN as one high-performing candidate among several nonlinear models for this dataset.^[18–26]^

Chronological validation in a later admission period provides a more demanding assessment than a random hold-out because it preserves calendar order and exposes the models to a lower outcome prevalence. However, development and temporal cohorts still came from the same hospital, electronic medical record, and clinical workflow. Independent multicenter and prospective validation are therefore necessary to determine generalizability across institutions, populations, bilirubin testing practices, and information systems.

### 4.6. Strengths of this study

This study has several strengths. First, it spans approximately 10 years and includes 986 neonates. Second, the hemolytic-disease variable was completely excluded before retraining rather than merely omitted from presentation. Third, model stability was assessed by repeated stratified 5-fold cross-validation repeated 5 times, and locked models were evaluated in a later temporal cohort. Fourth, a record-level timestamp audit confirmed that every retained predictor preceded outcome ascertainment. Fifth, peak total bilirubin and other outcome-derived variables were excluded from model inputs. Finally, performance evaluation included discrimination, bootstrap CIs, calibration, Brier score, DCA, locked-threshold metrics, and repeated grouped permutation importance.

### 4.7. Limitations

Several limitations remain. First, this retrospective study was conducted at one center. Although chronological validation used a later admission period, it remains an internal temporal validation within the same clinical system and cannot establish external transportability. The marked prevalence difference between periods also indicates a calendar-time distribution shift. Second, retrospective data remain susceptible to selection bias, measurement bias, and unmeasured confounding. Third, several clinically relevant predictors were unavailable, including feeding status, postnatal weight loss, maternal-neonatal blood-group incompatibility, DAT results, prior phototherapy, genetic factors, and longitudinal bilirubin trajectories. Future datasets should incorporate these variables and enable direct benchmarking against American Academy of Pediatrics hour-specific bilirubin risk assessment.^[27–31]^

Fourth, CK and LDH were complete and routinely collected in this cohort but may be selectively ordered elsewhere. Fifth, the hemolytic-disease field had an irreconcilable coding conflict and was therefore completely excluded; the revised model cannot quantify hemolysis-related risk. Sixth, the fixed analytical outcome threshold of 230 µmol/L does not replace age- and gestation-specific treatment thresholds and may limit clinical comparability. Seventh, grouped permutation importance provides global association, not causation or patient-specific explanation. Finally, the extremely high performance of several nonlinear models requires cautious interpretation and confirmation in prospectively collected, clinically representative external cohorts.

### 4.8. Future directions

Future research should prioritize external multicenter validation, prospective evaluation, and comparison with guideline-based risk assessment. Datasets from different regions and hospital levels should test calibration, threshold transportability, and performance with and without institution-specific laboratory markers. Prospective studies should determine whether model-assisted screening improves bilirubin monitoring without causing unnecessary testing or treatment. Future models should incorporate longitudinal bilirubin trends, feeding status, postnatal weight change, treatment data, blood-group and DAT results, and genetic or hemolytic markers before any electronic decision-support implementation is considered.

## 5. Conclusion

After complete exclusion of the hemolytic-disease variable, the TabPFN-based model showed stable repeated-cross-validation performance and strong temporal discrimination, probability accuracy, clinical net benefit, and threshold-dependent performance. Random forest achieved marginally higher temporal discrimination, whereas TabPFN had the lowest Brier score and achieved 100% sensitivity and NPV at the development-derived threshold. The leading retained predictors were maternal disease count, abortion or preterm history, postnatal abnormality, and birth-weight category. These results support TabPFN as a promising candidate model but not as a clinically deployable or universally superior tool. Independent multicenter validation, prospective evaluation, and benchmarking against standard-of-care risk assessment are required before clinical use.

## Author contributions

**Conceptualization:** Yu Zhang.

**Data curation:** Yu Zhang.

**Formal analysis:** Yu Zhang.

**Funding acquisition:** Yu Zhang.

**Investigation:** Yu Zhang.

**Methodology:** Yu Zhang.

**Project administration:** Hui Wang, Liping Zhang.

**Resources:** Hui Wang, Liping Zhang.

**Software:** Hui Wang, Liping Zhang.

**Supervision:** Hui Wang, Liping Zhang.

**Validation:** Hui Wang, Liping Zhang.

**Visualization:** Hui Wang.

**Writing – original draft:** Hui Wang.

**Writing – review & editing:** Hui Wang.
